# Supplementary material for: Update of a prediction model for postoperative shoulder stiffness after arthroscopic rotator cuff repair
Source: Commun Med (Lond). 2025 Oct 2;5:413. doi: 10.1038/s43856-025-01125-w (PMC12491454; doi:10.1038/s43856-025-01125-w)
Supplement: Supplementary file 2 — Supplementary Information [file 43856_2025_1125_MOESM2_ESM.pdf]

Supplementary Information

Update of a prediction model for postoperative shoulder stiffness after arthroscopic rotator cuff repair

Thomas Stojanov, Soheila Aghlmandi, Andreas Marc Müller, Philipp Moroder, Alexandre Lädermann, Cornelia Baum, ARCR_Pred Study Group, Laurent Audigé

1 Table of Contents

2 METHODS 2

2.1 PARTICIPANTS FOLLOW-UPS 2
2.2 DELPHI SURVEY DETAILS 2
Supplementary Table 1: Surgeon-level characteristics distribution 2
Supplementary File 1. Delphi survey round 1 4
Supplementary File 2. Delphi survey round 2 12
2.3 STATISTICAL ANALYSIS 16
2.3.1 Objectives 16
2.3.2 Sample size calculation 16
2.3.3 Outcome 16
2.3.4 Handling of missing data 16
2.3.5 Descriptive statistics and univariable regression analysis 16
2.3.6 Type of model 17
2.3.7 First model version summary 17
2.3.8 Prediction modeling 17
2.3.8.2 Model validation indicators 18
2.4 PREDICTION TOOL 19

3 RESULTS 20

Supplementary Table 2. Baseline diagnostic-related variables and their association with the occurrence of post-operative shoulder stiffness 21
Supplementary Table 3. Operation patient-related variables distribution and their association with the occurrence of post-operative shoulder stiffness 22
Supplementary File 3. TRIPOD+AI Checklist 23

4 SUPPLEMENTARY REFERENCES 25

This supplemental material has been provided by the authors to report additional details.

2 METHODS

2.1 Participants follow-ups

Study procedures were described in the study protocol.[1] Inherent to data collection in the field of observational studies, a variation in the effective duration exists between the completion of the various clinical examinations and questionnaires. All baseline variables were collected using the baseline case report form (CRF) or using the patient-reported questionnaire before the surgery. The median time between questionnaire completion was 1 day, ranging from immediate to 165 days post-baseline. Between baseline examination and surgery, the median duration was 8 days, ranging from 0 to 177 days. The median number of days between the operation performance and the completion of the 6-month examination was 183 days, ranging from 117 to 314 days. The median number of days between the 6-month clinical examination and the questionnaire completion was 1 day, ranging from 0 to 365 days. We addressed this issue by considering "missing" the data that were collected outside of the following intervals: baseline data (0 – 180 days pre-surgery); and 6-month data (112 – 280 days post-surgery). Data from N = 8 patients were then considered missing at the 6-month follow-up.

2.2 Delphi survey details

Audience

We invited the 53 experienced surgeons (Supplementary Table 1) who performed at least one surgery in the context of the ARCR_Pred study[1] to fill out a *2 rounds* questionnaire between October 2021 and January 2022. The questionnaire was prepared using a REDCap database, an electronic data capture system embedding web-based survey tools.[2]

Supplementary Table 1: Surgeon-level characteristics distribution

Years of Training/Practice in Shoulder Surgery	1-20	>20-50	>50-100	>100	Total
1-5 years	13	6	5	0	**23**
>5-10 years	0	7	1	4	**11**
>10-20 years	0	1	9	4	**14**
>20 years	0	0	4	1	**5**
**Total**	**12**	**13**	**19**	**9**	**53**

First online survey

Agreement with the POSS-definition

In the first survey (Supplemental File 1), surgeons were asked to indicate their degree of agreement (using a 5-item Likert scale to indicate their degree of agreement (1. "I fully agree", 2. "I agree", 3. "I do not know", 4. "I disagree", 5. "I fully disagree")) with the definition below, as well as provide any comments that may contribute to an improvement of the definition.

A previous consensus definition of POSS had been developed by shoulder surgeons who were involved in a Delphi process to define a core event set of ARCR[3]. This definition remained unpublished at the time, however, was further adapted and approved by another surgeon panel in the context of shoulder arthroplasty[4]. We defined POSS as a composite outcome (i.e. meeting at least one of the following conditions):

1. Any restriction in passive range of motion occurring at least 3 months after the operation implying a modification in the usual patient's care (e.g. physiotherapy, medication, or an intervention requiring manipulation under anesthesia), or:

2. A persisting post-operative restriction at 6 months in passive motion in at least two planes (flexion, abduction, and external rotation in zero-degree abduction). The assessment of the Restriction in range of motion has been done separately for each plane: Flexion: total motion inferior or equal to 90 degrees or gleno-humeral motion (fixed scapula) inferior or equal to 80 degrees. Abduction: total motion inferior or equal to 80 degrees or gleno-humeral motion (fixed scapula) inferior or equal to 60 degrees. External rotation in zero-degrees abduction: gleno-humeral (fixed scapula) motion inferior or equal to 20 degrees or inferior to 50% of the contralateral side value.

Prognostic factors value

Surgeons were then asked to rank the prognostic importance of potential prognostic factors on a 5-item Likert scale (1: "Not important" to 5: "Highly important") relying on their clinical expertise. We listed 53 potential prognostic factors collected during baseline, operation, or 6-week examinations including 22 that were identified via systematic literature review[5] and 31 identified via the screening ARCR_Pred database by the core project team (TS, LA and AMM). We provided open fields to report on additional potential factors that we may have omitted from the list, even if these factors would not be documented in the context of the ARCR_Pred study.

Supplementary File 1. Delphi survey round 1

ARCR_pred: prognostic factors for post-operative shoulder stiffness

Dear colleague,

Thank you for taking part in this survey. You will find all the important definitions on the following pages.

Please make sure that you answer all questions and complete the survey before leaving.

Kind regards

Thomas Stojanov
PhD student
thomas.stojanov@usb.ch

Introduction

Postoperative shoulder stiffness (POSS) is one of the most frequent adverse event after an arthroscopic rotator cuff repair (ARCR). We would like to be able to prevent its occurrence by developing and using clinical prediction models, which is one of the main objective of our ARCR_pred study.

The choice of factors specifying such models should rely on the literature and on expert assessment. We therefore systematically reviewed the evidence published since 2014 and identified 22 potential prognostic factors. We complemented these findings with 31 factors identified in other reviews and preliminary analyses.

We will ask you to rank the prognostic importance of each factor according to your personal knowledge of the literature and your clinical expertise.

All the surgeons who performed at least one operation in our project can take part in this survey.

Project funded by Swiss National Science Fundation (SNSF) and Swiss Orthopaedics, and coordinated by University Hospital Basel, BS and Schulthess Klinik, ZH

Swiss National Science Foundation   swiss orthopaedics

Prognostic factor definition

"A prognostic factor is any variable, that, among people with a given health condition (i.e. a startpoint), is associated with (the risk of) a subsequent clinical outcome (i.e. an endpoint)."(Richard D Riley, Prognosis Research in Healthcare, Concepts, Methods, and Impact, ed. Oxford University Press 2019)

Example: Fatty infiltration is known as a prognostic factor for re-tear after an ARCR.

Outcome definition

Post operative shoulder stiffness (POSS) was defined as an adverse event of the deep soft tissue event group accompanied by restrictions in passive range of motion (see attached publication published in the context of shoulder arthroplasty).

[Attachment: "2019-Audigé-JSES-Core set of unfavorable events of SA.pdf"]

In the present survey, we defined POSS as a composite outcome (i.e. meeting at least one of the following conditions):

Any restriction in passive range of motion occurring at least 3 months after the operation implying a modification in the usual patient's care (e.g. physiotherapy, medication or an intervention requiring manipulation under anesthesia), or:

A persisting post-operative restriction at 6 months in passive motion in at least two planes (flexion, abduction and ext. rotation in 0 degree abduction).

Restriction is assessed separately for each plane:
Flexion: total motion inferior or equal to 90 degrees or gleno-humeral motion (fixed scapula) inferior or equal to 80 degrees
Abduction: total motion inferior or equal to 80 degrees or gleno-humeral motion (fixed scapula) inferior or equal to 60 degrees
External rotation in 0 degrees abduction: gleno-humeral (fixed scapula) motion inferior or equal to 20 degrees or inferior to 50% of the contralateral side value.

Do you agree with this definition?	◯ I fully agree<br>◯ I agree<br>◯ I do not know<br>◯ I disagree<br>◯ I fully disagree
Why do not you agree?	______________________________
Any comment related to the use of this definition?	______________________________

Patient-related factors

According to your knowledge and your experience as a clinician, can you please rank the following patient-related factors regarding their prognostic importance from "No prognostic importance" up to "High prognostic importance" for the occurrence of POSS?

If you think that a factor has a high prognostic importance for the occurrence of a POSS, the factor should be ranked as "Highly important".

If you think that a factor has no prognostic importance for the occurrence of a POSS, the factor should be ranked as "Not important".

	Not important	Weakly important	Moderately important	Important	Highly important
Age	○	○	○	○	○
Alcohol drinks	○	○	○	○	○
ASA classification	○	○	○	○	○
Body Mass Index	○	○	○	○	○
Chronic pulmonary disease	○	○	○	○	○
Depression or anxiety	○	○	○	○	○
Diabetes	○	○	○	○	○
Dyslipidemia	○	○	○	○	○
Hyper-hypothyroidism	○	○	○	○	○
Hypercholesterolemia	○	○	○	○	○
Hypertension	○	○	○	○	○
Preoperative level of sport activities	○	○	○	○	○
Relatives with diabetes	○	○	○	○	○
Relatives with stiffness	○	○	○	○	○
Sex	○	○	○	○	○
Smoking status	○	○	○	○	○
Systematic lupus erythematosus	○	○	○	○	○
Vitamin D deficiency	○	○	○	○	○

Do you have other patient-level factors in mind that should be considered?
○ Yes
○ No

What is your suggestion?

((e.g. I think the socio-economic status is associated with POSS))

Disease-related factors

According to your knowledge and your experience as a clinician, can you please rank the following disease-related factors regarding their prognostic importance from "Not important prognostic factor" up to "Highly important prognostic factor" for the occurrence of POSS?

If you think that a factor has a high prognostic importance for the occurrence of a POSS, the factor should be ranked as "Highly important".

If you think that a factor has no prognostic importance for the occurrence of a POSS, the factor should be ranked as "Not important".

	Not important	Weakly important	Moderately important	Important	Highly important
Acromiohumeral distance	○	○	○	○	○
Affected side dominant	○	○	○	○	○
Critical shoulder angle	○	○	○	○	○
Fatty infiltration of repaired tendons	○	○	○	○	○
Involvement of infraspinatus	○	○	○	○	○
Involvement of subscapularis	○	○	○	○	○
Involvement of supraspinatus	○	○	○	○	○
Osteoarthritis	○	○	○	○	○
Preoperative functional status	○	○	○	○	○
Preoperative shoulder stiffness (i.e. restriction in passive range of motion)	○	○	○	○	○
Symptom duration	○	○	○	○	○
Tear size (in anteroposterior dimension)	○	○	○	○	○
Traumatic onset	○	○	○	○	○
Tear severity	○	○	○	○	○
Tendon degeneration	○	○	○	○	○
Tendon delamination	○	○	○	○	○
Tendon retraction	○	○	○	○	○

Do you have other disease-related factors in mind that should be considered?
○ Yes
○ No

What is your suggestion?

____
((e.g. I think the number of muscles involved is associated with POSS))

05/23/2023 1:47pm projectredcap.org REDCap

Procedure-related factors

According to your knowledge and your experience as a clinician, can you please rank the following procedure-related factors regarding their prognostic importance from "Not important prognostic factor" up to "Highly important prognostic factor" for the occurrence of POSS? If you think that a factor has a high prognostic importance for the occurrence of a POSS, the factor should be ranked as "Highly important". If you think that a factor has no prognostic importance for the occurrence of a POSS, the factor should be ranked as "Not important".

	Not important	Weakly important	Moderately important	Important	Highly important
Acromioclavicular joint resection	◯	◯	◯	◯	◯
Acromioplasty	◯	◯	◯	◯	◯
Biceps procedures	◯	◯	◯	◯	◯
Capsulotomy	◯	◯	◯	◯	◯
Number of anchors	◯	◯	◯	◯	◯
Number of threads	◯	◯	◯	◯	◯
Preoperative medication	◯	◯	◯	◯	◯
Preoperative physiotherapy	◯	◯	◯	◯	◯
Preoperative steroid infiltration	◯	◯	◯	◯	◯
Operation duration	◯	◯	◯	◯	◯
Repair technique (e.g. single-row, double-row or transoessous-equivalent)	◯	◯	◯	◯	◯

Do you have other procedure-related factors in mind that should be considered?

◯ Yes
◯ No

What is your suggestion?

____
((e.g. I think the experience of the surgeon is associated with POSS))

Early post-operative management

According to your knowledge and your experience as a clinician, can you please rank the following early post-operative management factors regarding their prognostic importance from "Not important prognostic factor" up to "Highly important prognostic factor" for the occurrence of POSS? If you think that a factor has a high prognostic importance for the occurrence of a POSS, the factor should be ranked as "Highly important". If you think that a factor has no prognostic importance for the occurrence of a POSS, the factor should be ranked as "Not important".

	Not important	Weakly important	Moderately important	Important	Highly important
Immobilization duration	○	○	○	○	○
Immobilization position (using aid)	○	○	○	○	○
Pain level at 6 weeks	○	○	○	○	○
Passive motion at 6 weeks	○	○	○	○	○
Procedure type (i.e. none, physiotherapy, watertherapy, ergotherapy, or other rehabilitation procedure)	○	○	○	○	○
Time of starting passive range of motion	○	○	○	○	○
Time of starting active range of motion	○	○	○	○	○

Do you have other early post-operative management factors in mind that should be considered?
○ Yes
○ No

What is your suggestion?

____
((i.e. I think the intensity of the physiotherapy is associated with POSS))

05/23/2023 1:47pm projectredcap.org REDCap

General comments

Any general comment?	○ Yes<br>○ No
What is your comment?	______________________________

Second survey

The surgeons who replied to the first round were invited to fill out a second survey. After analyzing the results of the first survey, responding surgeons received a feedback comparing their rating with the average and confidence interval (see the example for AMM in Supplemental File 2). Surgeons were then asked if they wanted to revise their rating. In that second survey, surgeons also could rate the additional potential prognostic factors that were suggested by the panel in the first round.

Supplementary File 2. Delphi survey round 2

REDCap

ARCR_Pred-Delphi-POSS-PF-02
Record ID 26 (Mueller A)

Survey

Response is only partial and is not complete. Response was added on 01/04/2022 2:30pm.

Record ID	26

Introduction

During the first Delphi process round, we asked you if you agreed with our first outcome definition and to rank the prognostic importance of each factor according to your personal knowledge of the literature and your clinical expertise. Overall, 53 surgeons were invited, 44 of you fully responded.

It corresponds to a response rate of 83%. We would like to thank you for your great involvement.

We will present you the first Delphi process round results and aim to reach a consensus about the prognostic factor rankings.

Project funded by Swiss National Science Fundation (SNSF) and Swiss Orthopaedics, and coordinated by University Hospital Basel, BS and Schulthess Klinik, ZH

Swiss National Science Foundation swiss orthopaedics

Outcome definition

As a remainder, during the first Delphi process round, we defined post-operative shoulder stiffness (POSS) as a composite outcome *(i.e. meeting at least one of the following conditions)*:

1. Any restriction in passive range of motion occurring at least 3 months after the operation implying a modification in the usual patient's care *(e.g. physiotherapy, medication or an intervention requiring manipulation under anesthesia)*, or:
2. A persisting post-operative restriction at 6 months in passive motion in at least two planes *(flexion, abduction and ext. rotation in 0 degree abduction)*.

   Restriction is assessed separately for each plane:
   Flexion: total motion inferior or equal to 90 degrees or gleno-humeral motion (fixed scapula) inferior or equal to 80 degrees
   Abduction: total motion inferior or equal to 80 degrees or gleno-humeral motion (fixed scapula) inferior or equal to 60 degrees
   External rotation in 0 degrees abduction: gleno-humeral (fixed scapula) motion inferior or equal to 20 degrees or inferior to 50% of the contralateral side value.

Overall, 39 (88%) of you agreed with the above cited definition.
Yet, three respondents (7%) suggested a minor change: a shift from 3 to 6 months for the first outcome condition.

This point will be addressed in the project.

Any comment related to the use of this definition?

Second round Delphi process

Our objective with this project is to reach a consensus regarding the prognostic factors rankings, we will therefore compare your gradings (in red) to the average gradings (mean and standard deviations in blue) reported by all the ARCR_Pred surgeons.

After this comparison, we will ask you if you want to change your gradings.
As a remainder, the question asked was:

According to your knowledge and your experience as a clinician, can you please rank the following factors regarding their prognostic importance from *"No prognostic importance"* up to *"High prognostic importance"* for the occurrence of POSS?

If you think that a factor has a high prognostic importance for the occurrence of a POSS, the factor should be ranked as "Highly important".

If you think that a factor has no prognostic importance for the occurrence of a POSS, the factor should be ranked as "Not important".

Patient-related factors

Mean importance level grading and standard deviation (blue dots and lines) along with your own grading (red dots) for patient-related factors

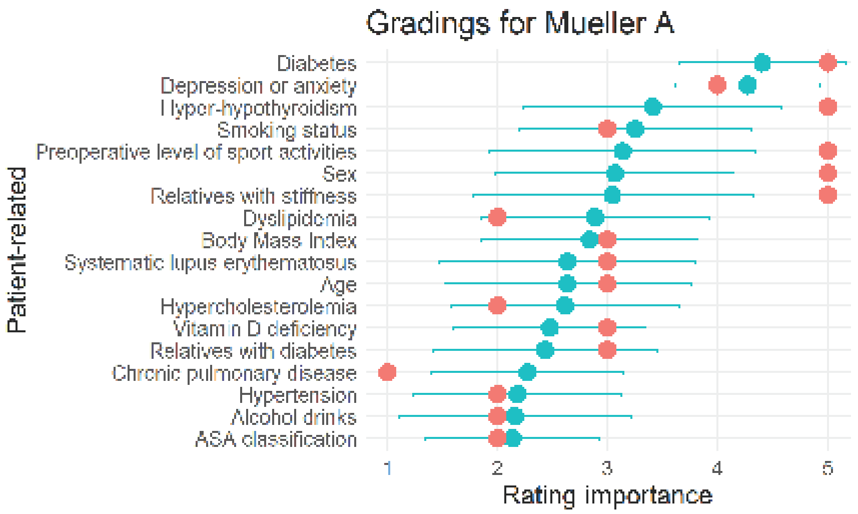

In view of these results and your previous ratings, would you like to revise your rating for any of the listed factors?

* must provide value

No, I confirm all my previous ratings   Yes, I want to revise one or more ratings

Do you have any comment?

Disease-related factors

Mean importance level grading and standard deviation (blue dots and lines) along with your own grading (red dots) for disease-related factors

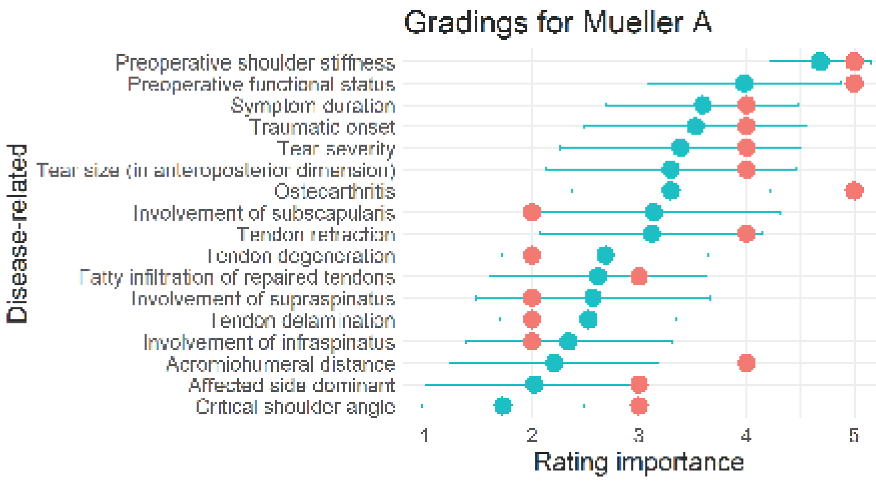

In view of these results and your previous ratings, would you like to revise your rating for any of the listed factors?

* must provide value

No, I confirm all my previous ratings   Yes, I want to revise one or more ratings

Do you have any comment?

Procedure-related factors

Mean importance level grading and standard deviation (blue dots and lines) along with your own grading (red dots) for procedure-related factors

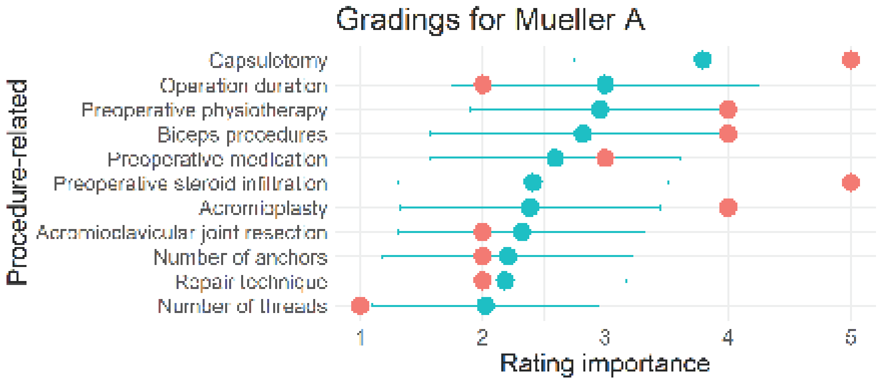

In view of these results and your previous ratings, would you like to revise your rating for any of the listed factors?
* must provide value

No, I confirm all my previous ratings Yes, I want to revise one or more ratings

Do you have any comment?

Early post-operative management

Mean importance level grading and standard deviation (blue dots and lines) along with your own grading (red dots) for patient-related factors

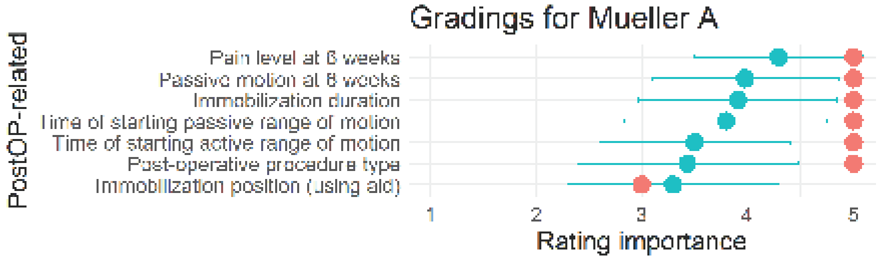

In view of these results and your previous ratings, would you like to revise your rating for any of the listed factors?
* must provide value

No, I confirm all my previous ratings Yes, I want to revise one or more ratings

Do you have any comment?

Suggested factors

Can you please rate these additional parameters suggested by participants at the end of the first survey?

According to your knowledge and your experience as a clinician, can you please rank the following patient-related factors regarding their prognostic importance from *"No prognostic importance"* up to *"High prognostic importance"* for the occurrence of POSS?

If you think that a factor has a high prognostic importance for the occurrence of a POSS, the factor should be ranked as "Highly important".

If you think that a factor has no prognostic importance for the occurrence of a POSS, the factor should be ranked as "Not important".

Suggested factors

	Not important	Weakly important	Moderately important	Important	Highly important
**Accuracy of patient expectations** * must provide value	○	○	○	○	●
**Bone quality** * must provide value	○	●	○	○	○

**Complex Regional Pain Syndrome at 6 weeks** * must provide value	○	○	○	○	●
**Employment status** * must provide value	○	○	○	○	●
**History of shoulder stiffness on contralateral side** * must provide value	○	○	○	○	●
**Hyperlaxity** * must provide value	○	●	○	○	○
**Operation fear** * must provide value	○	○	○	●	○
**Pain level the day after surgery** * must provide value	○	○	○	●	○
**Physical therapist experience** * must provide value	○	●	○	○	○
**Post-operative pain medication and dosage at 6 weeks** * must provide value	○	○	○	●	○
**Preoperative capsulitis partial tears (PASTA)** * must provide value	○	○	○	○	●
**Preoperative type of activity at work** * must provide value	○	○	○	○	●
**Preoperative work physical commitment** * must provide value	○	○	○	○	●
**Retear at 6 weeks** * must provide value	○	○	●	○	○
**Rotator interval synovitis** * must provide value	○	○	○	●	○
**Socioeconomic status** * must provide value	○	○	○	●	○
**Vitamin D deficiency at 6 weeks** * must provide value	○	●	○	○	○
**Worker's compensation claim status at 6 weeks** * must provide value	○	○	○	○	●

Do you have any comment?

General comments

Any general comment? * must provide value

○ Yes
● No

Form Status

Complete? Incomplete

2.3 Statistical analysis

2.3.1 Objectives

Our objective is to update and validate a model predicting the occurrence of post-operative shoulder stiffness by patients undergoing an arthroscopic rotator cuff repair in Switzerland using the ARCR_Pred data.

2.3.2 Sample size calculation

According to ARCR_Pred study protocol written in 2018, we accounted for the minimum of ten events per variable (EPV) to allow for the inclusion of a maximum of ten predictors in the model predicting POSS [6,7]. The estimated event rate for shoulder stiffness from our pilot dataset is 8.3%, however because of the retrospective data collection and some inherent judgement in recording this event, we suspect it was underestimated. Our personal experience tells us that at least 10% of patients will be affected, which has been considered to reach the required EPV. The sample size calculation was performed to reach satisfying precision for both primary outcomes; the higher resulting number determines the final number of patients to be recruited. This approach led to a sample of 920 and 900 patients for change of OSS in 6 months and shoulder stiffness, respectively. Anticipating a maximum 5% dropout rate at 6 months (based on our personal experience) leads to the inclusion of 970 patients with surgical repair. All baseline and operation variables were considered as potential prognostic factor for inclusion in a related model.

2.3.3 Outcome

The definition suggested in the Delphi process (see Delphi survey details section) was used as main outcome. It corresponds to a dichotomized composite outcome that can occur up to 6 months after the surgery.

2.3.4 Handling of missing data

We expected the dataset to have variable missing data rates and that these missing values were missing at random. We therefore considered the use of multiple imputation using chained equations (MICE package) [8] and the aregImpute() function from the rms package[9], with a number of datasets that depended on the highest missingness rate observed for a specific variable (e.g. if age is missing in 10% of the cases, then 10 multiple imputed datasets will be used). A sentitivity analysis was performed to compare multiple imputation (MI) results to the results of the complete-case (CC) analysis and was reported at each analysis phase.

2.3.5 Descriptive statistics and univariable regression analysis

Continuous and discrete variables were described using median and interquartile ranges (IQR), while categorical variables were expressed with numbers and percentages. Whenever variables had categories representing less

than 5% of the overall patients, regrouping variables adjacent categories was considered only if it makes clinical sense, otherwise, variables were dropped before modeling phase. More information regarding the decision for each variable is available in the Supplementary Material X. The missing rate and prognostic value of each variable was reported. Univariable logistic regression models were also estimated between each baseline and operation variable and the outcome. Odds ratios and their 95% confidence intervals (CI) were reported.

2.3.6 Type of model

A logistic regression was used to model the dichotomized outcome.

2.3.7 First model version summary

In 2021, Audigé et al. developed the first version of a model (called in the present manuscript KWS-POSS)[10] using the Schulthess Klinik register documenting patients undergoing ARCR at a single orthopaedic center.[11] In this study, authors described 1330 ARCR, of them, 112 (8.3%) experienced a POSS. They assessed the predictive ability of 29 potential prognostic factors. Their final model was composed of 8 baseline and operation-related variables (Male sex β = -0.92 CI (-1.112 to -0.273), Overweight, β = -0.256 CI (-0.668 to 0.155), Pre-operative passive abdution (10 degrees unit), β = -0.091 CI (-0.161 to -0.022), Traumatic onset, β = -0.053 CI (0.473 to 0.367), Tear severity: Partial tear (ref), Single full tear, β = −0.549 (-1.036 to -0.061), Two or 3 tendons (only 1 full tear) β = −0.618 (-1.238 to 0.002), Massive tear (2 or 3 full tears), β = -0.533 (-1.177 to 0.110), Tendon degeneration β = −0.346 (-0.765 to 0.073), Acromioplasty, β = −0.534 (-1.121 to 0.052) and Capsulotomy, β = −0.036 CI (-0.695 to 0.623)). This model had a moderate predictive ability with an AUC of 0.67. No data were reported regarding the calibration accuracy of this model.

2.3.8 Prediction modeling

2.3.8.1.1 External validation

In a first step, we externally validated the model KWS-POSS[10] with original regression coefficients. AUC, calibration in the large and calibration slope were assessed on the new data. In a second step, we reestimated the coefficients of the model KWS-POSS using the ARCR_Pred data.

After performing each surgery, the operating surgeon was also asked to predict the risk of occurrence of POSS for the operated patient. The predictive ability of this prediction was also compared to observed data in terms of discrimination and calibration.

2.3.8.1.2 Internal validation

In a second step, we developed and internally validated, using bootstrapping, new prediction models. We firstly identified the so-called “Expert” model, which was composed of the 7 parameters with the highest predictive ability. In parallel, we performed a Lasso variable selection procedure to identify the variables with the highest predictive ability, this model was called “LASSO”. A third model, “Expert-reduced” was developed after application of a LASSO variable selection on the “Expert” model. A fourth model, “Combined” was finally developed after combining the information of both models. A fifth model, “Combined - Penalized” was developed after identification of the optimal penalty term – supposed to reduce overfitting, using the pentrace() function from the rms package.[9,12]

2.3.8.2 Model validation indicators

Each developed model was assessed in terms of model performance. Steyerberg et al. described an ABCD for model validation that was used for the following four sub-sections.[13]

2.3.8.2.1.1 A: Alpha: Calibration-in-the-large

Calibration refers to the agreement between observed endpoints and predictions.[14] A graphical representation of the model can be used by plotting the predicted POSS against the observed proportion of the outcome. Imperfect calibration can then be characterized by deviations from these ideal values. The intercept A will then relate to calibration-in-the-large that will compare the mean of all predicted risks with the mean of observed risk. This parameter will then highlight whether predicted risks are too high, or too low, in comparison to the observed risks.

2.3.8.2.1.2 B: Beta: Calibration slope

If a calibration slope (B) is different than 1, it indicates that predictions are too extreme: low predictions are too low and high predictions are too high.

2.3.8.2.1.3 C: Concordance statistic: discrimination

Discrimination refers to the ability of the model to distinguish a patient with the outcome (POSS) from a patient without (no POSS). In our case, we used the area under the receiver operating characteristic (ROC) curve, which represents the Sensitivity (true-positive rate) against 1 – Specificity (false-positive rate) for consecutive cut-offs for the predicted risks.

2.3.8.2.1.4 D: Decision-curve analysis and planned model use

Clinical usefulness was defined as the ability to make better decisions with a model than without.[14,15] Our main objective with this project was to target patients at high risk of POSS using a clinical prediction model. If the type of variables selected in the model allow for it, the first aim is to identify variables that could be maximized by clinicians (e.g. better baseline range of motion is associated with less POSS after the surgery: then clinicians could advocate to maximize this value prior the surgery). The second aim was to advocate for extra-treatment (e.g. more intensive physiotherapy post-surgery) in case the patients have a higher risk of occurrence of POSS.

2.4 Prediction tool

For presentation and didactic purposes, we developed a R Shiny app highlighting the use of the finally reported prediction model. The R Shiny app presents individualized probabilities of occurrence of POSS varying according to a given combination of patient, diagnostic and treatment values that depended on model specification.

3 RESULTS

Supplementary Table 2. Baseline diagnostic-related variables and their association with the occurrence of post-operative shoulder stiffness

				Complete-case analysis (N = 833)		Multiple imputation (M = 10)
**Baseline variables (N = 41)**		**Missing**	**PV**	**Distribution**	**Univariable regression**	**Univariable regression**
**Diagnostic-related factors (N = 11)**						
Dominant affected side, n (%)			1.93	590 (71)	1.12 (0.73 to 1.76)	1.07 (0.7 to 1.64)
Symptom duration of more than 6 months, n (%)			3.61	394 (47)	1.12 (0.76 to 1.65)	1.03 (0.71 to 1.49)
Traumatic onset, n (%)			3.43	442 (53)	1.01 (0.69 to 1.48)	1.08 (0.76 to 1.54)
**X-Ray findings**						
Acromiohumeral distance (in mm) (0 - 20), Median (IQR)		3.1	2.05	10 (9 – 11)	0.87 (0.8 to 0.94)	0.88 (0.81 to 0.96)
Critical Shoulder Angle (in °) (20 - 50), Median (IQR)		2.9	1.73	34 (32 – 37)	1.02 (0.97 to 1.07)	1.02 (0.97 to 1.07)
Mild osteoarthritis, n (%)		2.8	3.36	97 (12)	0.8 (0.39 to 1.48)	1.05 (0.59 to 1.88)
**MRI findings**						
Highest degree of fatty infiltration (repaired tendons), n (%)			2.59			
	Stage 0			429 (52)	Ref.	Ref.
	Stage 1			277 (33)	1.03 (0.67 to 1.58)	1.1 (0.72 to 1.66)
	Stage 2			115 (14)	1.41 (0.8 to 2.42)	1.34 (0.79 to 2.27)
	Stage 3-4			12 (1.4)	0.54 (0.03 to 2.77)	0.44 (0.06 to 3.44)
Supraspinatus involvement, n (%)			2.52			
	Intact tendon			45 (5.4)	Ref.	Ref.
	Partial tear			193 (23)	1.76 (0.66 to 6.14)	1.72 (0.58 to 5.06)
	Full-thickness tear			595 (71)	1.84 (0.73 to 6.22)	1.75 (0.61 to 5.04)
Infraspinatus involvement, n (%)			2.3			
	Intact tendon			493 (59)	Ref.	Ref.
	Partial tear			197 (24)	1.35 (0.85 to 2.13)	1.23 (0.78 to 1.95)
	Full-thickness tear			143 (17)	1.39 (0.82 to 2.28)	1.36 (0.83 to 2.21)
Subscapularis involvement, n (%)			3.16			
	Intact tendon			407 (49)	Ref.	Ref.
	Partial tear			297 (36)	1.1 (0.72 to 1.66)	1.05 (0.71 to 1.56)
	Full-thickness tear			129 (15)	0.91 (0.49 to 1.6)	0.83 (0.48 to 1.44)
Tear severity, n (%)		0.1	3.3			
	Partial tear			175 (21)	Ref.	Ref.
	Single full tear			176 (21)	0.61 (0.33 to 1.11)	0.61 (0.35 to 1.08)
	Two or three tendons (only one full)			401 (48)	0.82 (0.51 to 1.32)	0.83 (0.52 to 1.31)
	Massive tear			81 (9.7)	1.29 (0.65 to 2.47)	1.23 (0.66 to 2.31)

Abbreviations: IQR: Interquartile Range; PV: Prognostic Value. **Note**. The baseline variables, categorized into diagnostic-related factors are examined. For each variable, the table presents the proportion of missing values, the prognostic value (PV) indicating the results of the Delphi survey invovling 44 surgeons (high the PV is, higher the surgeons estimated this specific variable was important) and the distribution of the variable. In addition, the provided table compares the results of complete-case analysis and multiple imputation univariable regression with the occurrence of post-operative shoulder stiffness.

Supplementary Table 3. Operation patient-related variables distribution and their association with the occurrence of post-operative shoulder stiffness

				Complete-case analysis (N = 833)		Multiple imputation (M = 10)
**Operation variables (N = 12)**		**Missing**	**PV**	**Distribution**	**Univariable regression**	**Univariable regression**
**Operation findings**						
Tear severity, n (%)			3.3			
	Partial tear			112 (13)	Ref.	Ref.
	Single full tear			218 (26)	0.56 (0.3 to 1.06)	0.81 (0.46 to 1.42)
	Two or three tendons (only one full)			367 (44)	0.65 (0.38 to 1.16)	0.79 (0.46 to 1.35)
	Massive tear			136 (16)	0.62 (0.31 to 1.24)	0.89 (0.47 to 1.67)
Tendon degeneration, n (%)			2.59	325 (39)	0.86 (0.57 to 1.3)	1.08 (0.75 to 1.56)
Tendon delamination, n (%)			2.45	284 (34)	1.05 (0.69 to 1.6)	1.16 (0.79 to 1.7)
**Repair surgical details**						
Number of anchors used, Median (IQR)			2.09	3.00 (2.00 – 4.00)	0.96 (0.84 to 1.1)	0.99 (0.89 to 1.12)
Supraspinatus repair technique, n (%)			2.18			
	Double row			181 (22)	Ref.	Ref.
	Not repaired			88 (11)	1.52 (0.72 to 3.16)	1.23 (0.64 to 2.36)
	Single row			157 (19)	1.75 (0.95 to 3.28)	1.28 (0.74 to 2.2)
	TOE with knots			282 (34)	1.33 (0.76 to 2.4)	1.18 (0.72 to 1.92)
	TOE without knots			125 (15)	0.62 (0.26 to 1.38)	0.52 (0.24 to 1.11)
Infraspinatus repair technique, n (%)			2.18			
	Double row			103 (12)	Ref.	Ref.
	Not repaired			495 (59)	0.87 (0.49 to 1.65)	0.75 (0.45 to 1.27)
	Single row			73 (8.8)	1.65 (0.75 to 3.62)	1.16 (0.56 to 2.4)
	TOE without knots			49 (5.9)	0.67 (0.21 to 1.85)	0.47 (0.17 to 1.31)
	TOE with knots		2.18	113 (14)	0.63 (0.27 to 1.44)	0.54 (0.26 to 1.14)
Subscapularis repair technique, n (%)						
	Double row			28 (3.4)	Ref.	Ref.
	Not repaired			471 (57)	4.17 (0.86 to 75.02)	2.4 (0.47 to 12.17)
	Single row			269 (32)	4.58 (0.93 to 82.83)	2.67 (0.52 to 13.65)
	TOE without knots			6 (0.7)	27 (2.6 to 660.66)	12.95 (1.35 to 124.29)
	TOE with knots			59 (7.1)	2.5 (0.38 to 49.17)	1.35 (0.23 to 7.89)
**Additional procedures**						
Acromioclavicular joint resection, n (%)			2.25	63 (7.6)	1.59 (0.79 to 3)	1.53 (0.81 to 2.88)
Acromioplasty, n (%)			2.25	507 (61)	0.69 (0.46 to 1.04)	0.72 (0.49 to 1.05)
Capsulotomy, n (%)			3.82	37 (4.4)	1.86 (0.77 to 3.99)	1.68 (0.77 to 3.68)
Biceps status and treatment, n (%)			2.86			
	Abnormal (tendinopathy)			623 (75)	Ref.	Ref.
	Normal			124 (15)	1.03 (0.58 to 1.75)	0.93 (0.56 to 1.53)
	Ruptured or already treated			86 (10)	0.38 (0.13 to 0.87)	0.59 (0.27 to 1.3)
Operation duration (in 10-minutes unit), Median (IQR)			2.95	7.5 (5.5 – 10.0)	1.08 (1.02 to 1.14)	1.08 (1.03 to 1.15)

Abbreviations: IQR: Interquartile Range; PV: Prognostic Value; TOE: Transosseous equivalent. **Note**. The operation variables are examined. For each variable, the table presents the proportion of missing values, the prognostic value (PV) indicating the results of the Delphi survey invovling 44 surgeons (high the PV is, higher the surgeons estimated this specific variable was important) and the distribution of the variable. In addition, the provided table compares the results of complete-case analysis and multiple imputation univariable regression with the occurrence of post-operative shoulder stiffness.

Supplementary File 3. TRIPOD+AI Checklist

TRIPOD+AI

Version: 11-January-2024

Section/Topic	Item	Development / evaluation[1]	Checklist item	Reported on page
**TITLE**				
Title	1	D;E	Identify the study as developing or evaluating the performance of a multivariable prediction model, the target population, and the outcome to be predicted	1
**ABSTRACT**				
Abstract	2	D;E	See TRIPOD+AI for Abstracts checklist	
**INTRODUCTION**				
Background	3a	D;E	Explain the healthcare context (including whether diagnostic or prognostic) and rationale for developing or evaluating the prediction model, including references to existing models	8
	3b	D;E	Describe the target population and the intended purpose of the prediction model in the context of the care pathway, including its intended users (e.g., healthcare professionals, patients, public)	8
	3c	D;E	Describe any known health inequalities between sociodemographic groups	
Objectives	4	D;E	Specify the study objectives, including whether the study describes the development or validation of a prediction model (or both)	8-9
**METHODS**				
Data	5a	D;E	Describe the sources of data separately for the development and evaluation datasets (e.g., randomised trial, cohort, routine care or registry data), the rationale for using these data, and representativeness of the data	10
	5b	D;E	Specify the dates of the collected participant data, including start and end of participant accrual; and, if applicable, end of follow-up	10
Participants	6a	D;E	Specify key elements of the study setting (e.g., primary care, secondary care, general population) including the number and location of centres	10
	6b	D;E	Describe the eligibility criteria for study participants	10
	6c	D;E	Give details of any treatments received, and how they were handled during model development or evaluation, if relevant	
Data preparation	7	D;E	Describe any data pre-processing and quality checking, including whether this was similar across relevant sociodemographic groups	10 / Suppl.
Outcome	8a	D;E	Clearly define the outcome that is being predicted and the time horizon, including how and when assessed, the rationale for choosing this outcome, and whether the method of outcome assessment is consistent across sociodemographic groups	11
	8b	D;E	If outcome assessment requires subjective interpretation, describe the qualifications and demographic characteristics of the outcome assessors	11
	8c	D;E	Report any actions to blind assessment of the outcome to be predicted	
Predictors	9a	D	Describe the choice of initial predictors (e.g., literature, previous models, all available predictors) and any pre-selection of predictors before model building	11-12
	9b	D;E	Clearly define all predictors, including how and when they were measured (and any actions to blind assessment of predictors for the outcome and other predictors)	
	9c	D;E	If predictor measurement requires subjective interpretation, describe the qualifications and demographic characteristics of the predictor assessors	11-12. Supp
Sample size	10	D;E	Explain how the study size was arrived at (separately for development and evaluation), and justify that the study size was sufficient to answer the research question. Include details of any sample size calculation	Suppl.
Missing data	11	D;E	Describe how missing data were handled. Provide reasons for omitting any data	Suppl.
Analytical methods	12a	D	Describe how the data were used (e.g., for development and evaluation of model performance) in the analysis, including whether the data were partitioned, considering any sample size requirements	12 / Suppl.
	12b	D	Depending on the type of model, describe how predictors were handled in the analyses (functional form, rescaling, transformation, or any standardisation).	
	12c	D	Specify the type of model, rationale[2], all model-building steps, including any hyperparameter tuning, and method for internal validation	13
	12d	D;E	Describe if and how any heterogeneity in estimates of model parameter values and model performance was handled and quantified across clusters (e.g., hospitals, countries). See TRIPOD-Cluster for additional considerations[3]	
	12e	D;E	Specify all measures and plots used (and their rationale) to evaluate model performance (e.g., discrimination, calibration, clinical utility) and, if relevant, to compare multiple models	13-14
	12f	E	Describe any model updating (e.g., recalibration) arising from the model evaluation, either overall or for particular sociodemographic groups or settings	13
	12g	E	For model evaluation, describe how the model predictions were calculated (e.g., formula, code, object, application programming interface)	13 / Suppl.
Class imbalance	13	D;E	If class imbalance methods were used, state why and how this was done, and any subsequent methods to recalibrate the model or the model predictions	
Fairness	14	D;E	Describe any approaches that were used to address model fairness and their rationale	
Model output	15	D	Specify the output of the prediction model (e.g., probabilities, classification). Provide details and rationale for any classification and how the thresholds were identified	14

[1] D=items relevant only to the development of a prediction model; E=items relating solely to the evaluation of a prediction model; D;E=items applicable to both the development and evaluation of a prediction model
[2] Separately for all model building approaches.
[3] TRIPOD-Cluster is a checklist of reporting recommendations for studies developing or validating models that explicitly account for clustering or explore heterogeneity in model performance (eg, at different hospitals or centres). Debray et al, BMJ 2023; 380: e071018 [DOI: 10.1136/bmj-2022-071018]

Page 1 of 2

Training versus evaluation	16	D;E	Identify any differences between the development and evaluation data in healthcare setting, eligibility criteria, outcome, and predictors	
Ethical approval	17	D;E	Name the institutional research board or ethics committee that approved the study and describe the participant-informed consent or the ethics committee waiver of informed consent	
**OPEN SCIENCE**				
Funding	18a	D;E	Give the source of funding and the role of the funders for the present study	
Conflicts of interest	18b	D;E	Declare any conflicts of interest and financial disclosures for all authors	
Protocol	18c	D;E	Indicate where the study protocol can be accessed or state that a protocol was not prepared	
Registration	18d	D;E	Provide registration information for the study, including register name and registration number, or state that the study was not registered	
Data sharing	18e	D;E	Provide details of the availability of the study data	
Code sharing	18f	D;E	Provide details of the availability of the analytical code[4]	
**PATIENT & PUBLIC INVOLVEMENT**				
Patient & Public Involvement	19	D;E	Provide details of any patient and public involvement during the design, conduct, reporting, interpretation, or dissemination of the study or state no involvement.	
**RESULTS**				
Participants	20a	D;E	Describe the flow of participants through the study, including the number of participants with and without the outcome and, if applicable, a summary of the follow-up time. A diagram may be helpful.	15
	20b	D;E	Report the characteristics overall and, where applicable, for each data source or setting, including the key dates, key predictors (including demographics), treatments received, sample size, number of outcome events, follow-up time, and amount of missing data. A table may be helpful. Report any differences across key demographic groups.	Suppl.
	20c	E	For model evaluation, show a comparison with the development data of the distribution of important predictors (demographics, predictors, and outcome).	Suppl.
Model development	21	D;E	Specify the number of participants and outcome events in each analysis (e.g., for model development, hyperparameter tuning, model evaluation)	Suppl.
Model specification	22	D	Provide details of the full prediction model (e.g., formula, code, object, application programming interface) to allow predictions in new individuals and to enable third-party evaluation and implementation, including any restrictions to access or re-use (e.g., freely available, proprietary)[5]	Suppl.
Model performance	23a	D;E	Report model performance estimates with confidence intervals, including for any key subgroups (e.g., sociodemographic). Consider plots to aid presentation.	Table 1
	23b	D;E	If examined, report results of any heterogeneity in model performance across clusters. See TRIPOD Cluster for additional details[3].	
Model updating	24	E	Report the results from any model updating, including the updated model and subsequent performance	
**DISCUSSION**				
Interpretation	25	D;E	Give an overall interpretation of the main results, including issues of fairness in the context of the objectives and previous studies	20
Limitations	26	D;E	Discuss any limitations of the study (such as a non-representative sample, sample size, overfitting, missing data) and their effects on any biases, statistical uncertainty, and generalizability	21
Usability of the model in the context of current care	27a	D	Describe how poor quality or unavailable input data (e.g., predictor values) should be assessed and handled when implementing the prediction model	
	27b	D	Specify whether users will be required to interact in the handling of the input data or use of the model, and what level of expertise is required of users	
	27c	D;E	Discuss any next steps for future research, with a specific view to applicability and generalizability of the model	21-22

From: Collins GS, Moons KGM, Dhiman P, et al. *BMJ* 2024;385:e078378. doi:10.1136/bmj-2023-078378

[4] This relates to the analysis code, for example, any data cleaning, feature engineering, model building, evaluation.
[5] This relates to the code to implement the model to get estimates of risk for a new individual.

4 SUPPLEMENTARY REFERENCES

1. Audige L, Bucher HCC, Aghlmandi S, et al. Swiss-wide multicentre evaluation and prediction of core outcomes in arthroscopic rotator cuff repair: protocol for the ARCR_Pred cohort study. *BMJ Open* 2021; **11**(4): e045702.
2. Harris PA, Taylor R, Minor BL, et al. The REDCap consortium: Building an international community of software platform partners. *Journal of biomedical informatics* 2019; **95**: 103208.
3. Audige L, Flury M, Muller AM, ARCR CES Consensus Panel, Durchholz H. Complications associated with arthroscopic rotator cuff tear repair: definition of a core event set by Delphi consensus process. *J Shoulder Elbow Surg* 2016; **25**(12): 1907-17.
4. Audigé L, Schwyzer H-K, Äärimaa V, et al. Core set of unfavorable events of shoulder arthroplasty: an international Delphi consensus process. *J Shoulder Elb Surg* 2019; **28**(11): 2061-71.
5. Stojanov T, Modler L, Muller AM, et al. Prognostic factors for the occurrence of post-operative shoulder stiffness after arthroscopic rotator cuff repair: a systematic review. *BMC Musculoskelet Disord* 2022; **23**(1): 99.
6. Peduzzi P, Concato J, Kemper E, Holford TR, Feinstein AR. A simulation study of the number of events per variable in logistic regression analysis. *J Clin Epidemiol* 1996; **49**(12): 1373-9.
7. Hemingway H, Croft P, Perel P, et al. Prognosis research strategy (PROGRESS) 1: a framework for researching clinical outcomes. *BMJ* 2013; **346**: e5595.
8. Van Buuren S, Groothuis-Oudshoorn K. mice: Multivariate imputation by chained equations in R. *Journal of statistical software* 2011; **45**: 1-67.
9. Harrell FE. Regression modeling strategies: with applications to linear models, logistic regression, and survival analysis: Springer; 2001.
10. Audigé L, Aghlmandi S, Grobet C, et al. Prediction of shoulder stiffness after arthroscopic rotator cuff repair. *Am J Sports Med* 2021; **49**(11): 3030-9.
11. Flury M, Kolling C, Grobet C, Kunz SN, Audigé L. Implementation of a local outcome register for arthroscopic rotator cuff tear repair. *Obere Extremität* 2015; **10**(1): 33-40.
12. Van Calster B, van Smeden M, De Cock B, Steyerberg EW. Regression shrinkage methods for clinical prediction models do not guarantee improved performance: Simulation study. *Statistical Methods in Medical Research* 2020; **29**(11): 3166-78.
13. Steyerberg EW, Vergouwe Y. Towards better clinical prediction models: seven steps for development and an ABCD for validation. *European Heart Journal* 2014; **35**(29): 1925-31.
14. Steyerberg EW, Vickers AJ, Cook NR, et al. Assessing the performance of prediction models: a framework for traditional and novel measures. *Epidemiology* 2010; **21**(1): 128-38.
15. Vickers AJ, Holland F. Decision curve analysis to evaluate the clinical benefit of prediction models. *Spine J* 2021; **21**(10): 1643-8.
