# Supplementary material for: Update of a prediction model for postoperative shoulder stiffness after arthroscopic rotator cuff repair
Source: Commun Med (Lond). 2025 Oct 2;5:413. doi: 10.1038/s43856-025-01125-w (PMC12491454; doi:10.1038/s43856-025-01125-w)
Supplement: Supplementary file 3 — Description of Additional Supplementary Files [file 43856_2025_1125_MOESM3_ESM.pdf]

Description of Additional Supplementary Files

File name: Supplementary Data 1

Description: Variables list, description, data management and reason for missingness

File name: Supplementary Data 2

Description: Baseline patient-related variables distribution and their association with the occurrence of post-operative shoulder stiffness
