# Supplementary material for: Update of a prediction model for postoperative shoulder stiffness after arthroscopic rotator cuff repair
Source: Commun Med (Lond). 2025 Oct 2;5:413. doi: 10.1038/s43856-025-01125-w (PMC12491454; doi:10.1038/s43856-025-01125-w)
Supplement: Supplementary file 6 — Reporting Summary [file 43856_2025_1125_MOESM6_ESM.pdf]

nature portfolio

Corresponding author(s): Thomas Stojanov

Last updated by author(s): Apr 15, 2025

Reporting Summary

Nature Portfolio wishes to improve the reproducibility of the work that we publish. This form provides structure for consistency and transparency in reporting. For further information on Nature Portfolio policies, see our Editorial Policies and the Editorial Policy Checklist.

Statistics

For all statistical analyses, confirm that the following items are present in the figure legend, table legend, main text, or Methods section.

n/a	Confirmed	
☐	☒	The exact sample size (*n*) for each experimental group/condition, given as a discrete number and unit of measurement
☐	☒	A statement on whether measurements were taken from distinct samples or whether the same sample was measured repeatedly
☐	☒	The statistical test(s) used AND whether they are one- or two-sided<br>*Only common tests should be described solely by name; describe more complex techniques in the Methods section.*
☐	☒	A description of all covariates tested
☐	☒	A description of any assumptions or corrections, such as tests of normality and adjustment for multiple comparisons
☐	☒	A full description of the statistical parameters including central tendency (e.g. means) or other basic estimates (e.g. regression coefficient) AND variation (e.g. standard deviation) or associated estimates of uncertainty (e.g. confidence intervals)
☐	☒	For null hypothesis testing, the test statistic (e.g. *F*, *t*, *r*) with confidence intervals, effect sizes, degrees of freedom and *P* value noted<br>*Give P values as exact values whenever suitable.*
☒	☐	For Bayesian analysis, information on the choice of priors and Markov chain Monte Carlo settings
☐	☒	For hierarchical and complex designs, identification of the appropriate level for tests and full reporting of outcomes
☐	☒	Estimates of effect sizes (e.g. Cohen's *d*, Pearson's *r*), indicating how they were calculated

Our web collection on statistics for biologists contains articles on many of the points above.

Software and code

Policy information about availability of computer code

Data collection	*Provide a description of all commercial, open source and custom code used to collect the data in this study, specifying the version used OR state that no software was used.*
Data analysis	The statistical code produced to obtain the results was uploaded to Zenodo (Stojanov, T. (2024). Code for Update and validation of a model predicting the occurrence of postoperative shoulder stiffness after an arthroscopic rotator cuff repair in Switzerland. Zenodo. https://doi.org/10.5281/zenodo.11148522) and will be made available upon request.

For manuscripts utilizing custom algorithms or software that are central to the research but not yet described in published literature, software must be made available to editors and reviewers. We strongly encourage code deposition in a community repository (e.g. GitHub). See the Nature Portfolio guidelines for submitting code & software for further information.

Data

Policy information about availability of data

All manuscripts must include a data availability statement. This statement should provide the following information, where applicable:

- Accession codes, unique identifiers, or web links for publicly available datasets
- A description of any restrictions on data availability
- For clinical datasets or third party data, please ensure that the statement adheres to our policy

Following a period of embargo of at least two years after the end of the study in September 2024, metadata describing the type, size, and content of the dataset

will be published along with the study protocol on the open repository Zenodo (https://zenodo.org/). Researchers wishing to access the code and the full dataset will be able to file a request with the Data Access Committee of the Medical Faculty of the University of Basel (MF-DAC – email: med-dac@unibas.ch). The MF-DAC will act as an independent assessor of the request and grant access to the dataset if all ethical, legal, and scientific conditions are met.

Research involving human participants, their data, or biological material

Policy information about studies with human participants or human data. See also policy information about sex, gender (identity/presentation), and sexual orientation and race, ethnicity and racism.

Reporting on sex and gender	.
Reporting on race, ethnicity, or other socially relevant groupings	We don't control for ethinicity or race.
Population characteristics	Population characteristics are described in the Supplementary Information.
Recruitment	A cohort of 973 primary ARCR patients were prospectively enrolled in 18 Swiss and one German orthopedic clinics between June 2020 and November 2021 and followed up to 24 months postoperatively.
Ethics oversight	Ethics approval for the ARCR_Pred study was obtained on April 1st, 2020, from the lead ethics committee (EKNZ, Basel Switzerland; ID: 2019–02076, trial registration number NCT04321005). All participants provided informed written consent before enrollment in the study.

Note that full information on the approval of the study protocol must also be provided in the manuscript.

Field-specific reporting

Please select the one below that is the best fit for your research. If you are not sure, read the appropriate sections before making your selection.

☒ Life sciences ☐ Behavioural & social sciences ☐ Ecological, evolutionary & environmental sciences

For a reference copy of the document with all sections, see nature.com/documents/nr-reporting-summary-flat.pdf

Life sciences study design

All studies must disclose on these points even when the disclosure is negative.

Sample size	For the second primary outcome of shoulder stiffness, we accounted for a minimum of 10 events per variable to allow for the inclusion of a maximum of 10 predictors into the model.68 69 The estimated event rate for shoulder stiffness from our pilot data set was 8.3%, which according to our experience might reflect an underestimation of the true rate.17 Therefore, a 10% stiffness rate was assumed, which resulted in a sample size calculation of 900 patients.
Data exclusions	None
Replication	None
Randomization	None
Blinding	Not applicable

Reporting for specific materials, systems and methods

We require information from authors about some types of materials, experimental systems and methods used in many studies. Here, indicate whether each material, system or method listed is relevant to your study. If you are not sure if a list item applies to your research, read the appropriate section before selecting a response.

Materials & experimental systems

n/a	Involved in the study
☒	☐ Antibodies
☒	☐ Eukaryotic cell lines
☒	☐ Palaeontology and archaeology
☒	☐ Animals and other organisms
☐	☒ Clinical data
☒	☐ Dual use research of concern
☒	☐ Plants

Methods

n/a	Involved in the study
☒	☐ ChIP-seq
☒	☐ Flow cytometry
☒	☐ MRI-based neuroimaging

Clinical data

Policy information about clinical studies

All manuscripts should comply with the ICMJE guidelines for publication of clinical research and a completed CONSORT checklist must be included with all submissions.

Clinical trial registration: https://clinicaltrials.gov/study/NCT04321005

Study protocol: https://bmjopen.bmj.com/content/bmjopen/11/4/e045702.full.pdf

Data collection: 19 study centers in Switzerland and Germany

Outcomes: See protocol

Plants

Seed stocks: None

Novel plant genotypes: None

Authentication: None
